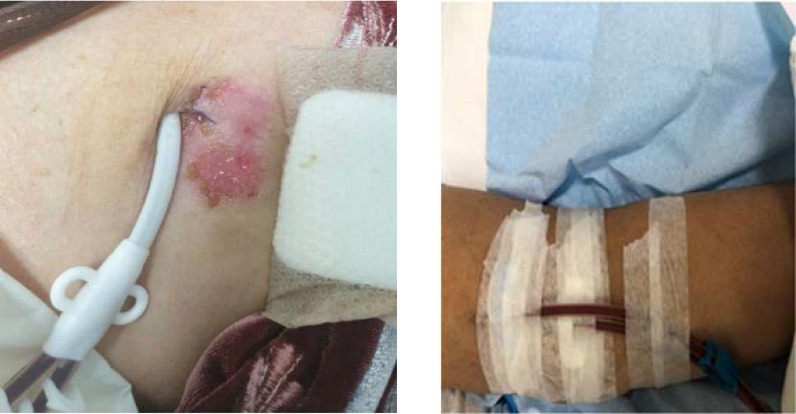# Contact dermatitis in hemodialysis patients at Al Wakra hospital

**DOI:** 10.5339/qmj.2022.fqac.18

**Published:** 2022-04-04

**Authors:** Ihab Elmadhoun, Saad Mahi, Rony Pulikkan, Ahmed Emam

**Affiliations:** ^1^Department of Nephrology, Al Wakra Hospital, Hamad Medical Corporation, Doha, Qatar E-mail: smahi@hamad.qa; ^2^Department of Internal Medicine, Al Wakra Hospital, Hamad Medical Corporation, Doha, Qatar

**Keywords:** Allergens, CD, HD patients

## Abstract

Background: Patients undergoing hemodialysis are exposed to various potential allergens from medication, dialysis catheters, topical antiseptics, and different adhesive dressings. Many patients develop a local allergic reaction and get itchy rashes, which may get infected, leading to significant morbidity and preventable health cost. In this study, we aimed to report the incidence of contact dermatitis (CD) and its potential complications in hemodialysis (HD) patients at the Al Wakra Hospital Dialysis unit.

Methods: We performed a retrospective chart review of documented local allergic reactions at vascular access sites for the HD patients at the Al Wakra Hospital.

Results: Currently, 102 patients are getting maintenance HD through catheters or arteriovenous (AV) fistula. Twelve (14.4%) patients developed CD (7 [58%] had cuffed jugular dialysis catheter, and 5 [42%] had an AV fistula). Most patients (75%) developed CD in the early period of dialysis initiation, and 25% developed it later in the course. Most patients responded to removing adhesive plasters and dressing the vascular access site using gauze only and topical steroids (hydrocortisone 1% cream/mometasone 0.1% cream). Two (16.6%) of the 12 patients developed vascular access site infection, of whom 1 had an AV fistula and developed a severe rash with cellulitis leading to sepsis and 2 admissions, although blood cultures remained negative. The patient responded to IV antibiotics and local mometasone 0.1% cream. Complete removal of all adhesive tapes helped prevent recurrence of the rash. Later, dressing of the AV fistula site was performed only with a cotton gauze. The second patient had a jugular catheter and developed an allergic rash leading to cellulitis and tunnel infection. Swab culture showed *Staphylococcus aureus* from the exit site sensitive to cloxacillin/cefazolin. The patient improved after local and oral antibiotics and removal of adhesive tapes. The catheter was not removed, and the patient did well.

Conclusion: The incidence of CD at our dialysis unit is 14.4%. Previously published reports from other dialysis units showed a lower incidence of 1.25%. Early identification and diagnosis of allergic rash at the vascular access site and avoidance of adhesive plasters and other potential allergens prevent complications like infection and loss of precious vascular accesses in these patients.

## Figures and Tables

**Figure 1.**